# Assessing the clinical value of microRNAs in formalin-fixed paraffin-embedded liposarcoma tissues: Overexpressed miR-155 is an indicator of poor prognosis

**DOI:** 10.18632/oncotarget.14320

**Published:** 2016-12-28

**Authors:** Nikolaos Kapodistrias, Konstantinos Mavridis, Anna Batistatou, Penelope Gogou, Vasilios Karavasilis, Ioannis Sainis, Evangelos Briasoulis, Andreas Scorilas

**Affiliations:** ^1^ Cancer Biobank Center, University of Ioannina, University Campus, Ioannina, Greece; ^2^ Department of Biochemistry and Molecular Biology, National and Kapodistrian University of Athens, Panepistimiopolis, Athens, Greece; ^3^ Department of Pathology, School of Medicine, University of Ioannina, Greece; ^4^ Clinical Oncology Department, Norwich University Hospital, UK; ^5^ London Sarcoma Service, Department of Oncology, UCLH, London, UK

**Keywords:** liposarcoma, miRNAs, miR-155, miRNA expression normalization, prognostic biomarker

## Abstract

Liposarcoma (LPS) is a malignancy with extreme heterogeneity and thus optimization towards personalizing patient prognosis and treatment is essential. Here, we evaluated miR-155, miR-21, miR-143, miR-145 and miR-451 that are implicated in LPS, as novel FFPE tissue biomarkers.

A total of 83 FFPE tissue specimens from primary LPS and lipomas (LPM) were analyzed. A proteinase K incubation-Trizol treatment coupled protocol was used for RNA isolation. After polyadenylation of total RNA and reverse transcription, expression analysis of 9 candidate reference and 5 target miRNAs was performed by qPCR. Genorm and NormFinder were used for finding the most suitable molecules for normalization. Survival analyses were performed in order to evaluate the prognostic potential of miRNAs.

MiR-103 and miR-191 are most suitable for normalization of miRNA expression in LPS. MiR-155 and miR-21 are clearly overexpressed (P<0.001) in LPS compared with LPM specimens, whereas miR-145 (P<0.001), miR-143 (P =0.008) and miR-451 (P=0.037) are underexpressed. MiR-155 (P=0.007) and miR-21 (P=0.029) are differentially expressed between well-differentiated, dedifferentiated, myxoid/round cell and pleomorphic LPs tumor subtypes. MiR-155 represents a novel independent indicator of unfavorable prognosis in LPS (HR = 2.97, 95% CI = 1.23–7.17, P = 0.016).

## INTRODUCTION

Liposarcoma (LPS) accounts for at least 20% of total soft tissue sarcomas cases [1, 2]. It is subdivided into four histologic subtypes forming three biologic groups with distinct morphological and cytogenetic characteristics: i) well-differentiated/ de-differentiated (WDLPS/DDLPS) with amplification of chromosome 12q13-1 resulting to *MDM2, HMGA2* and *CDK4* overexpression, ii) myxoid/round cell (MRC) LPS with translocation t(12;16)(q13;p11.2) leading to a uniquely aberrant transcription factor derived from FUS-CHOP fusion and iii) pleomorphic LPS, a high-grade, rare and aggressive disease variant [1–3].

Although each histological group has a different clinical behavior, treatment is largely the same for most LPS subtypes. Complete surgical resection of the tumor remains the cornerstone of primary treatment. Radiotherapy and conventional cytotoxic chemotherapy have a limited effect in confronting tumor spread, with the exception of MRC LPS, and their use remains controversial [1–5]. Guidelines for adjuvant and neoadjuvant therapy and the assessment of LPS histologic subtype fluctuate greatly, even among major sarcoma centers. Tumor grade, localization, size and subtype, are widely accepted prognostic factors in LPS [4, 6, 7]. Nonetheless, many LPS cases show a rapid recurrence rate and eventually progress to advanced non-manageable disease [7–9]. The prognostic and predictive value that is, primarily, based on simple morphological and cytogenic characteristics of the tumor may not be accurate, as it does not encompass the diverse underlying molecular mechanisms that drive LPS growth [3, 4, 8, 10]. Consequently, the identification of novel prognostic biomarkers is necessary and could help towards the stratification of patients into those who will ultimately benefit from (neo)adjuvant treatment, and those who can be spared from the harmful side-effects of cytotoxic therapy and can simply follow a monitoring approach [1, 7–10].

In this respect, the archives of formalin-fixed paraffin-embedded (FFPE) tissue biobanks represent an invaluable resource for the identification of novel cancer biomarkers, especially for malignancies with low prevalence, such as LPS. FFPE tissue samples are the most readily available and well documented archival material in pathology laboratories and tissue banks around the world [11]. However, FFPE tissues have not been widely used in gene expression profiling studies because formalin fixation can lead to largely degraded and chemically modified total RNA. Fortunately, this is not the case for microRNAs (miRNAs), a family of short RNA molecules that are resistant to both degradation and chemical modification and thus can be reliably measured in FFPE tissues [12, 13]. The holistic nature of clinical information that can be derived from miRNA expression analysis, since aberrant levels of a sole miRNA could reflect key-changes affecting a broad range of cancer-related biological pathways [14], as well as their rapid, easy, accurate and cost-effective determination *via* qPCR makes miRNAs uniquely valuable molecules in cancer biomarker research [13, 15, 16]. Successful examples of the clinical usefulness of miRNAs extracted from FFPE tissues include miR-194 and miR-210 which have been recently identified as robust prognostic biomarkers in clear cell renal cell carcinoma [17, 18].

Since 2002, when the very first report of miRNA deregulation, in chronic lymphocytic leukemia, took place by Calin *et al* in 2002 [19], the percentage of cancer-related studies implicating miRNAs as tumor-suppressors or oncogenes has risen a 1000-fold [16]. Even more impressive is the fact that several miRNAs are currently investigated as cancer biomarkers in > 100 clinical trials [15]. Current evidence have undoubtedly established the central involvement of miRNAs in the acquisition of aggressive tumor behavior, tumor progression and metastasis [14, 20]. Regarding LPS, there are reports - although limited compared to other malignancies - pointing out specific miRNAs that participate in liposarcomatogenesis and LPS tumor progression. MiR-155, miR-21 and miR-26a-2 are consistently reported to be severely upregulated in LPS compared to benign lipomatous tumors or normal fat. MiR-155, miR-26-a-2 and miR-135b have been described as oncogenes and key-regulators of LPS [21–27]. MiR-21 is the most widely known oncomiR participating in almost all human malignancies [24, 28–32]. Contrariwise, miR-145 and miR-143 form an anti-oncomir cluster which is down-regulated in most of the cancers and is able to inhibit tumorigenesis by targeting tumor-associated genes [33], and along with miR-451 have been described as tumor suppressors in most of the human malignancies studied [34], including LPS [35, 36]. MiR-26-a-2 and miR-135b are the only miRNAs up to date reported as LPS biomarkers and have both been associated with unfavorable prognosis [21, 27].

The aim of the present study was to provide information about the clinical utility of miRNAs participating in LPS initiation and progression, using a unique FFPE tissue sample cohort.

## RESULTS

### The combination of miR-103 and miR-191 levels is suitable for normalization of miRNA expression in liposarcoma

**Figure 1:**
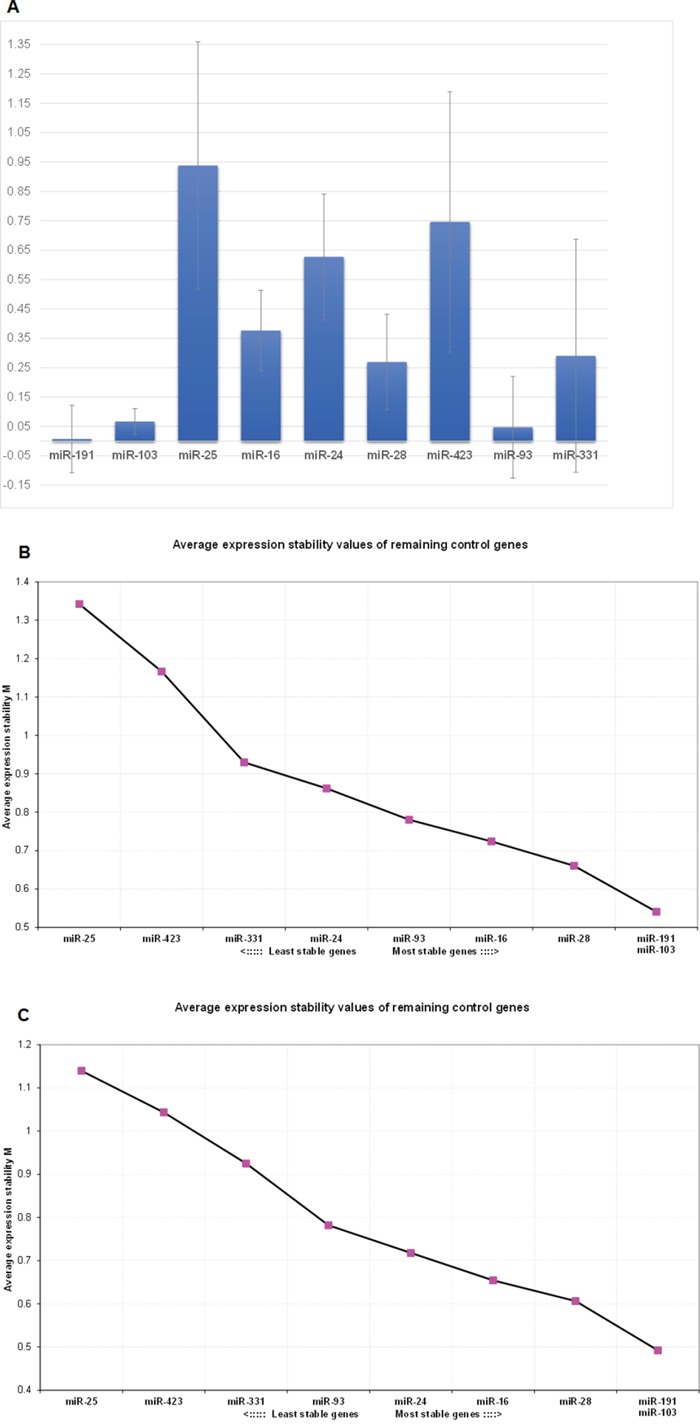
Selection of suitable reference miRNAs based on the combined NormFinderand GeNorm approach **Part A**: Graphical representation of the NormFinder output for selection of the best miRNAs to be used for normalization purposes. The inter-group variances are plotted and the error bars represent the average of the intra-group variances for each miRNA. **Part B**: GeNorm output in the same sample set used in the NormFinder analysis. The combination of miR-103 and miR-191 is suggested by the algorithm as the most stable pair. **Part C**: GeNorm output when including only LPS samples. The combination of miR-103 and miR-191 is again identified as the most stable pair for normalization.

The analysis of the expression of 9 candidate reference miRNA molecules in a set of 13 LPS and 9 LPM samples by the NormFinder algorithm (Table 1) identified miR-103 as the single most stable normalizer (stability value = 0.152) and miR-103 and miR-191 as the best combination (stability value = 0.127) for normalization of miRNA expression, taking into account both the stability of these molecules within LPS and within LPM (intragroup variation), as well as between LPS and LPM (intergroup variation). Indeed, as presented in Table 1, miR-103 and miR-191 are not only two of the most stable molecules, showing limited variation between LPS and LPM samples, but are also the most stable molecules in LPS samples (miR-103 LPS intragroup variation = 0.064, miR-191 LPS intragroup variation = 0.091). This is also depicted in Figure 1A, where bars represent inter-group variance and the error-bars represent the average of the intra-group variances. The ideal candidates, in our case miR-103 and miR-191, present an inter-group variation closer to zero than the other miRNAs and at the same time they have the smallest error bars compared to other molecules analyzed.

**Table 1:** NormFinder output containing, stability values, intra- and inter- group variation values and the suggestion of best miRNA and best combination of two genes given by the algorithm

miRNA	Stability value	Intragroup variation (LPM)	Intragroup variation(LPS)	Intragroupvariation
miR-191	0.153	0.139	0.091	0.007
miR-103	0.152	0.023	0.064	0.067
miR-25	1.098	0.034	0.809	0.938
miR-16	0.521	0.085	0.189	0.377
miR-24	0.787	0.083	0.345	0.627
miR-28	0.433	0.144	0.180	0.269
miR-423	0.969	0.319	0.567	0.746
miR-93	0.224	0.182	0.164	0.047
miR-331	0.504	0.087	0.707	0.290
**Best gene**	***miR-103***			
**Stability value**	0.152			
**Best combination of two genes**	***miR-191 and miR-103***			
**Stability value for best combination of two genes**	0.127			

The aforementioned results regarding the appropriateness of miR-103 and miR-191 combination for normalization were corroborated using the geNorm algorithm. As shown in Figure 1B, when using the same 22 LPS and LPM samples, miR-103 and miR-191 are indeed pointed out as the most stable combination of molecules. Results remain the same, when using only the LPS samples for analysis in an attempt to verify the low variability of miR-103 and miR-191 in LPS samples that was identified by NormFinder; this makes them ideal normalizers for liposarcoma miRNA expression analysis studies (Figure 1C).

### MiRNAs -155, -21, -145, -143 and -451 are differentially expressed between liposarcoma and lipoma samples

After selecting the best combination of reference genes for normalization and completing the quality control of the developed qPCR assays, a detailed expression analysis of the target miRNAs took place in LPS and LPM samples. As a first step of this analysis, significant differences in the expression levels of all 5 miRNA molecules were detected between LPS and LPM samples.

**Figure 2:**
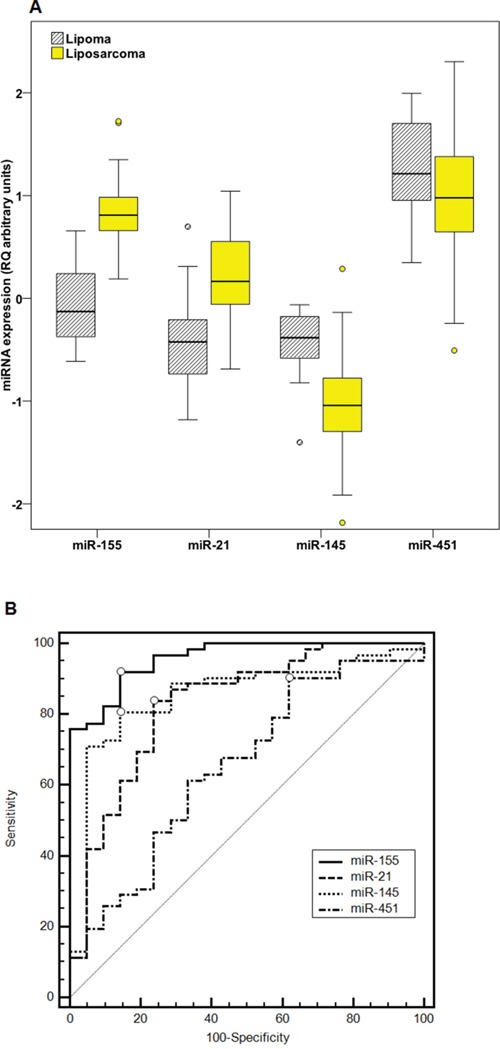
Differential expression of miRNAs between LPS and LPM samples **Part A**: Distribution of miR-155, miR-21, miR-145 and miR-451 expression (logarithmic values) between LPS and LPM samples. P values calculated by the Mann-Whitney *U* test. **Part B**: ROC curve analysis for miR-155, miR-21, miR-145 and miR-451 expression levels in liposarcoma and lipoma samples. Calculations according to DeLong *et al*. Points with the highest Youden index for each miRNA are marked with a circle.

MiR-155 and miR-21 were robustly upregulated in LPS compared with LPM samples (Figure 2A). More specifically, when comparing median expression, there was an 8.7–fold upregulation for miR-155 (P =4.3×10^-10^) and 3.9–fold for miR-21 (P= 7.0×10^-6^), respectively in LPS (median miR-155 expression = 5.12 RQ units, median miR-21 expression = 1.16 RQ units) compared with LPM samples (median miR-155 expression = 0.590 RQ units, median miR-21 expression = 0.299 RQ units).

Contrariwise, miR-145, miR-143 and miR-451 levels were downregulated in LPS compared with LPM samples. It should be noted that miR-143 levels were initially investigated in a subset of LPS and LPM tissue specimens (N = 18) and were found to be significantly downregulated (P = 0.008). Nonetheless, due to the very strong correlation observed between miR-143 and miR-145 levels (r_s_ = 0.968, P=4.6×10^-6^), as it may have been expected due to their co-cistronic expression, miR-143 was not evaluated further in the present study, because its expression analysis was expected to yield results very similar to that of miR-145. Indeed, miR-145 expression levels were significantly downregulated (P = 1.5×10^-6^) 4.6 times in LPS (median expression = 0.0720 RQ units) compared with LPM (median expression = 0.329 RQ units). MiR-451 was also downregulated in LPS (median expression = 7.54 RQ units) compared with LPM (median expression = 13.0 RQ units), but in a milder extent (1.7-fold, P = 0.037) (Figure 2A).

In an attempt to evaluate the differential diagnostic capacity of the miRNAs, ROC curves were developed and the AUC along with specificity and sensitivity values were calculated (Figure 2B and Table 2). Among the four miRNAs, miR-155 showed by far the best discriminatory value (AUC = 0.958, 95% CI = 0.918 – 0.998, Youden index J = 0.777, 95% CI = 0.645–0.871), followed by miR-145 (AUC = 0.853, 95% CI = 0.757 – 0.950, Youden index J = 0.664, 95% CI = 0.470–0.775). Notably, for miR-155 a combination of 91.9% sensitivity and 85.7% specificity can be achieved, whereas at fixed 95.0% sensitivity, specificity is 76.2% and at fixed specificity 95.0%, sensitivity is 77.4% (Table 2).

**Table 2:** Discriminatory capacity of miR-155, miR-21, miR-145, miR-451 for LPS *vs* LPM specimens as assessed by ROC curve analysis

miR-	AUC (95% CI)	P	Youden index J (95% CI)	Optimal Sensitivity, Specificity	Specificity at 90% fixed sensitivity (95% CI)	Specificity at 95% fixed sensitivity (95% CI)	Sensitivity at 90% fixed specificity (95% CI)	Sensitivity at 95% fixed specificity (95% CI)
miR-155	0.958 (0.918-0.998)	4.3×10^-10^	0.777 (0.645-0.871)	91.9%, 85.7%	85.71(57.14-95.24)	76.19(43.88-90.48)	82.26 (64.52-95.16)	77.42(61.29-90.32)
miR-21	0.829 (0.721-0.938)	7.0×10^-6^	0.600 (0.388-0.760)	83.9%, 76.2%	52.38(11.02-76.19)	38.1(9.52-61.90)	51.61(8.06-83.87)	41.94(4.84-69.35)
miR-145	0.853 (0.757-0.950)	1.5×10^-6^	0.664 (0.470-0.775)	80.6%, 85.7%	61.9(9.52-90.48)	23.81(0.00-71.43)	72.58(3.23-83.87)	70.97(6.45-86.18)
miR-451	0.653 (0.516-0.790)	0.037	0.284 (0.123-0.428)	90.3%, 38.1%	38.1(14.29-61.90)	23.81(0.00-52.38)	25.81(8.06-54.84)	19.35(3.23-37.10)

### MiR-155 and miR-21 present distinct expression patterns among different LPS tumor subtypes, and miR-155 is overexpressed in grade III tumors

**Figure 3:**
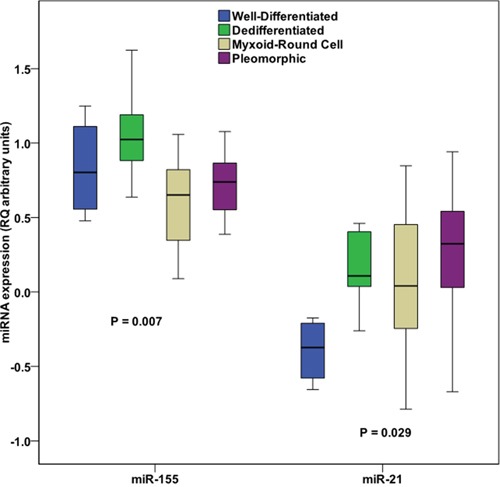
Distribution of miR-155 and miR-21 (logarithmic values) between different liposarcoma subtypes P values calculated by Kruskall-Wallis H test.

As presented in Figure 3, miR-155 (P = 0.007) and miR-21 (P = 0.029) expression levels were differentially expressed between well-differentiated, dedifferentiated, myxoid/round cell and pleomorphic LPs tumor subtypes. Regarding miR-21, the effect of the statistically significant differential distribution is attributed mainly to its low levels in well-differentiated tumors compared with other tumor subtypes (P = 0.011 for the comparison with dedifferentiated tumors, P = 0.026 for the comparison with myxoid/round cell tumors and P = 0.006 for the comparison with pleomorphic tumors). In the case of miR-155, its differential expression is reflected mainly in its high levels in the dedifferentiated compared to the myxoid/round cell (P = 2.1×10^-4^) and pleomorphic subtypes (P = 0.041).

Among the 4 miRNAs analyzed, only miR-155 showed an association with tumor grade (P = 0.006), as it was found to be overexpressed in grade III (median = 7.19 RQ units) compared with grade I/II tumors (median = 4.31 RQ units).

Increased age was weakly correlated with miR-21 levels in LPS patients (r_s_= 0.292, P = 0.021) and positively correlated with miR-155 levels in LPM patients (r_s_= 0.573, P = 0.008), whereas a negative correlation between age and miR-451 levels was also observed in LPM patients (r_s_= -0.548, P = 0.012). No other significant association was observed between miRNA expression and the remaining clinicopathological/demographic data of the study's patients.

### MiR-155 is associated with unfavorable oncologic outcome in liposarcoma patients

**Figure 4:**
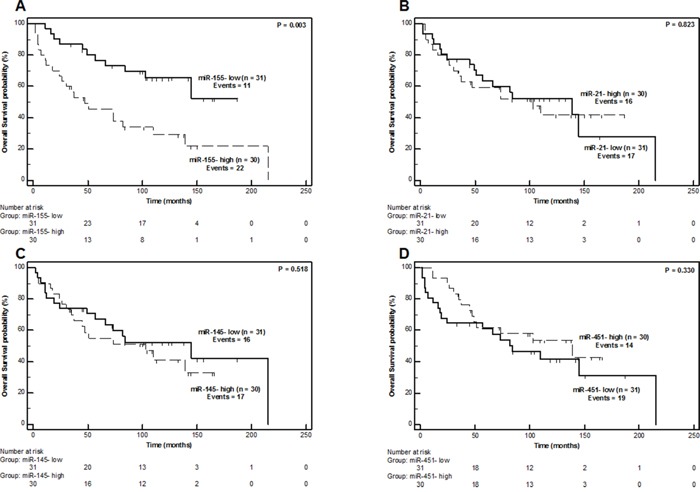
Overall Survival Kaplan-Meier Curves for miR-155 **(Part A)**, miR-21 **(Part B)**, miR-145 **(Part C)** and miR-451 **(Part D)** expression in liposarcoma patients. P values calculated by the log-rank algorithm.

As depicted in Figure 4, only miR-155 showed a statistically significant association (P = 0.003) with overall survival (OS). Higher miR-155 expression levels were associated with a worse OS course. Patients categorized as miR-155-high were evidently higher-risk individuals with a cumulative 5-year OS probability of 37.8 ± 9.2%, which is significantly lower than the corresponding 73.3 ± 8.1% probability of the miR-155-low individuals (Figure 4A). The Hazard Ratio (HR) for miR-155-high individuals was calculated by univariate Cox proportional hazard regression analysis to be 2.90 (95% CI = 1.39 – 6.03, P = 0.005).

**Figure 5:**
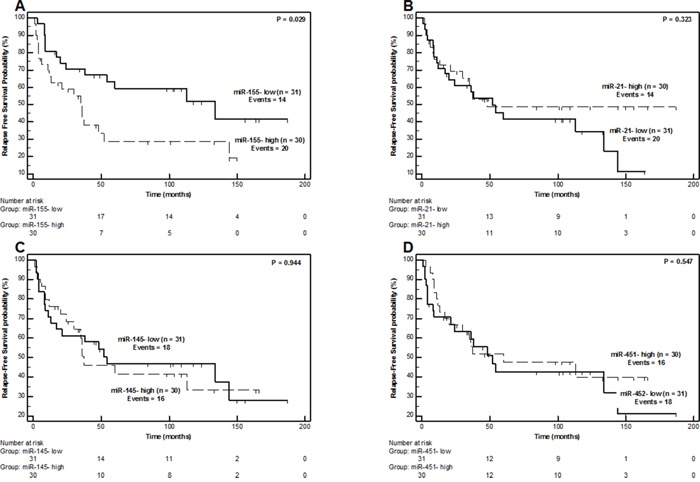
Relapse-free Survival Kaplan-Meier Curves for miR-155 **(Part A)**, miR-21 **(Part B)**, miR-145 **(Part C)** and miR-451 **(Part D)** expression in liposarcoma patients. P values calculated by the log-rank algorithm.

MiR-155 was also the only miRNA molecule of the study that was able to effectively scrutinize LPS patients according to their risk for disease relapse (local recurrence) (Figure 5). Individuals belonging to the miR-155-high category showed an inferior relapse-free survival (RFS) course (P = 0.029, Figure 5A), with a 5-year relapse-free probability of only 28.6 ± 9.3% compared to the respective 59.1 ± 9.2% probability of miR-155-low patients. Indeed, patients with high miR-155 levels were 2.11-times (95% CI = 1.06 –4.20) more likely to present local recurrence over time compared to miR-155-low individuals (P = 0.034).

Contrariwise to OS and RFS, no miRNA molecule was robustly associated with progression-free survival (PFS). MiR-155 expression showed a trend towards association with reduced PFS intervals, but not at a statistically significant extent (P = 0.209, Supplementary Figure 1A).

### MiR-155 represents a novel independent predictor of unfavorable prognosis in LPS

Tumors of grade III histology (HR = 2.29, 95% CI = 1.13 – 4.66, P = 0.022), larger tumor size (HR = 1.04, 95% CI = 1.00 – 1.08, P = 0.040), lesions of retroperitoneal localization (HR = 2.84, 95% CI = 1.26 – 6.41, P = 0.012) and patients with positive surgical margins (HR = 3.12, 95% CI = 1.47 – 6.59, P = 0.003) showed a significantly poorer prognosis with respect to OS outcome (Table 3). Thus, we corroborated the prognostic significance of these indicators in LPS showing the accuracy of sampling and classification of the patient group. Inferior RFS intervals could be more effectively predicted by retroperitoneal localization of LPS tumors (HR = 2.77, 95% CI = 1.28–5.97, P = 0.010) and positive surgical margins (HR = 3.16, 95% CI = 1.51–6.60, P = 0.002) (Table 4). It was imperative to test the prognostic value of miR-155 for independence from these robust and widely accepted prognostic factors. For this reason, multivariate Cox regression models were developed and it was found that miR-155 expression retains its prognostic significance for OS independently of currently accepted prognostic factors (Table 3). When correcting for grade, tumor size, tumor location, age, gender the HR for miR-155- high individuals was equal to 2.97 (95% CI = 1.23–7.17, P = 0.016. Results did not change when correcting for surgical margin status, instead of tumor location (HR for miR-155 = 5.18, 95% CI = 1.8–14.9, P =0.002) (Table 3).

**Table 3:** Cox proportional hazards regression analysis of miR-155 expression and clinicopathological variables for the prediction of overall survival

Covariant	Univariate Analysis			Multivariate Analysis		
	HR	95% CI	P*	HR	95% CI	P*
miR-155- high	2.90	1.39 – 6.03	0.005	2.97^a^5.18^b^	1.23–7.17^a^1.80-14.9^b^	0.016^a^0.002^b^
Grade III tumors	2.29	1.13–4.66	0.022	1.18^a^1.47^b^	0.514–2.72^a^0.546–3.93^b^	0.693^a^0.448^b^
Tumor size	1.04	1.00–1.08	0.040	1.04^a^1.06^b^	0.994–1.09^a^1.01–1.12^b^	0.089^a^0.026^b^
Retroperitoneal Location	2.84	1.26 –6.41	0.012	1.556^a^	1.01–2.41^a^	0.047^a^
Positive surgical margins	3.12	1.47–6.59	0.003	8.91^b^	3.32–23.92^b^	<0.001^b^
Age	1.01	0.987–1.03	0.409	1.01^a^1.03b	0.986–1.037^a^0.997–1.06^b^	0.395^a^0.076^b^
Gender (female)	1.42	0.705–2.86	0.326	0.954^a^1.16^b^	0.419–2.17^a^0.491–2.75^b^	0.912^a^0.733^b^

*Test for trend.

^a^Multivariate model adjusted for grade, tumor size, tumor location, age, gender.

^b^Multivariate model adjusted for grade, tumor size, surgical margins, age, gender.

HR: Hazard Ratio.

CI: Confidence Interval.

**Table 4:** Cox proportional hazards regression analysis of miR-155 expression and clinicopathological variables for the prediction of relapse-free survival

Covariant	Univariate Analysis			Multivariate Analysis		
	HR	95% CI	P*	HR	95% CI	P*
miR-155- high	2.11	1.06 –4.20	0.034	2.19^a^2.64^b^	0.987–4.86^a^1.06-6.62^b^	0.054^a^0.038^b^
Grade III tumors	1.93	0.976–3.81	0.059	1.41^a^1.99^b^	0.651–3.06^a^0.765–5.18^b^	0.383^a^0.159^b^
Tumor size	1.03	0.993–1.071	0.110	1.02^a^1.04^b^	0.972–1.07^a^0.988–1.09^b^	0.420^a^0.147^b^
Retroperitoneal Location	2.77	1.28–5.97	0.010	2.60^a^	1.09–6.19^a^	0.032^a^
Positive surgical margins	3.16	1.51–6.60	0.002	7.39^b^	2.78–19.6^b^	<0.001^b^
Age	1.01	0.992–1.04	0.204	1.01^a^1.03^b^	0.987–1.036^a^0.998–1.05^b^	0.379^a^0.065^b^
Gender (female)	0.697	0.339-1.43	0.326	0.954^a^0.530^b^	0.198–1.09^a^0.217–1.29^b^	0.076^a^0.163^b^

*Test for trend.

^a^Multivariate model adjusted for grade, tumor size, tumor location, age, gender.

^b^Multivariate model adjusted for grade, tumor size, surgical margins, age, gender.

HR: Hazard Ratio.

CI: Confidence Interval.

Regarding prediction of RFS, results were similar (Table 4); miR-155 expression could predict worse disease-free survival intervals, when adjusting for grade, tumor size, surgical margins, age, and gender (HR = 2.64, 95% CI = 1.06–6.62, P = 0.038), but statistical significance was marginal for miR-155 (HR = 2.19, 95% CI = 0.987–4.86, P = 0.054), when including tumor location instead of surgical margins in the multivariate model.

## DISCUSSION

Histologic grade, a more or less morphological feature, remains the most important prognostic factor for LPS patients. Individuals with distinct pathobiological features are erroneously categorized in the same prognostic group without taking into account the heterogeneity in the underlying molecular mechanisms that drive liposarcomatogenensis [2, 4, 7]. We hypothesized that miRNA expression analysis could address this issue [14–16].

Firstly, by testing several candidate normalizers, we concluded by using the geNorm and NormFinder algorithms [37–39] that the combination of miR-103 and miR-191 is most suitable for LPS miRNA expression normalization. In a recent study that used adipose tissues, including lipomas and normal adjacent adipose tissues, miR-103 was identified as the most stable miRNA [40], which is in fine agreement with our results. MiR-191 and miR-103 have been extensively used as normalizers in cancer-related miRNA expression studies [38, 41–47].

MiR-21 and miR-155 two of the most repeatedly reported upregulated miRNAs in LPS [23–26, 48] were also found to be robustly upregulated in our study and could efficiently discriminate LPS and LPM specimens with high specificity and sensitivity (Figure 2). Interestingly, a recent study by Boro *et al* showed that circulating levels of miRNA-155 in LPS patients correlate strongly with corresponding tissue levels. In the same study, miR-155 was identified as a powerful blood-based biomarker for the differential diagnosis of dedifferentiated LPS from healthy control, patients with other LPS subtypes, but also from benign lipoma cases [49]. Tissue miR-155 and miR-21 expression levels exhibited a distinct expression pattern among the various LPS subtypes in our study (Figure 3). More precisely, miR-21 presented higher levels in the dedifferentiated compared with other histological subtypes (Figure 3), in agreement with previously reported findings [35]. MiR-155 levels were also upregulated in the dedifferentiated compared to the myxoid/round cell and pleomorphic subtypes (Figure 3). MiR-155 was also overexpressed in higher grade tumors, in agreement with previous findings [25]. It has been previously reported that miR-451 could distinguish dedifferentiated liposarcoma and well-differentiated liposarcoma [50]; however here we did not observe this pattern for miR-451 expression.

Most importantly, miR-155 expression was found to be positively associated with unfavorable oncologic outcome in terms of OS (Figure 4A) and RFS course (Figure 5A). MiR-155 can provide important prognostic information (HR = 2.97, 95% CI = 1.23–7.17, P = 0.016), independently of currently used prognostic indicators, such as tumor grade, surgical margins, tumor size and tumor location. This holds true both for OS (Table 3) and RFS (Table 4). These results are in fine agreement with studies implicating miR-155 in LPS progression mechanisms [25, 26]. More precisely, the study of Zhang P. *et al* shows that miR-155 is associated with LPS tumor cell growth and colony formation *in vitro* and knockdown of its expression induces G1-S cell-cycle arrest. Blocking miR-155 expression in murine xenografts *in vivo*, results in significantly slower growth and decreased size of tumors. It is though that the oncogenic functions of miR-155 are exerted through casein kinase 1a (CK1a), which is a direct target of miR-155. An effect CK1a targeting by miR-155 is the enhanced b-catenin/cyclin D1 expression which ultimately promotes LPS proliferation and cell-cycle progression [26]. MiR-155 has also been associated with poor prognosis and acts as an oncomiR in several major human malignancies [51, 52], including breast cancer [53], lung cancer [31], colorectal cancer [30] and pancreatic cancer [29]. The only miRNAs that has been proven, up to now, to have prognostic value for LPS are miR-26a-2 and miR-135-b [21, 27]. MiR-26-a-2 expression targets *HOXA5* in LPS cells and results in resistance to apoptotic death *via* a p53-independent mechanism, whereas at the same time an association between miR-26-a-2 and LPS patient survival has also been observed [21, 22]. MiR-135b exerts its tumor promoting role in myxoid/round cell liposarcoma by supporting cell invasion and metastasis through suppressing thrombospondin 2 (THBS2). The diminished expression of *THBS2* leads to increased accumulation of matrix metalloproteinase 2 and ultimately influences cellular density and extracellular matrix structure, thereby resulting in tumor progression. MiR-135 has also been associated with poor prognosis in human myxoid liposarcoma [27].

On the contrary, miR-143, miR-145 and miR-451, molecules with tumor suppressor properties in LPS [23, 35, 36], are clearly downregulated in LPS compared to LPM tissue specimens (Figure 2), corroborating the results of previously published studies [23, 24, 35, 36, 48, 50]. MiR-143 inhibits proliferation, induces apoptosis and cytokinesis of dedifferentiated liposarcoma cells by lowering *BCL2*, *TOP2A*, *PRC1* and *PLK1* expression [36]. MiR-145 and miR-451 can also decrease cellular proliferation rate, prompt apoptosis and impair cell cycle progression [35]. MiR-145 and miR-143 are co-expressed in several tissues, since they are transcribed together from a cluster located on chromosome 5 (5q33), but have independent involvement in cellular processes [33]. In our study we confirmed this expected co-expression by observing a very strong correlation (r_s_= 0.968) between miR-143 and miR-145 expression levels. Despite their tumor suppressor properties in LPS, none of these molecules were able to predict the disease course of LPS patients in terms of neither OS, RFS, nor PFS in our study (Figure 4, Figure 5 and Supplementary Figure 1) and thus cannot be considered as prognostic biomarkers for LPS.

In conclusion, we show that miR-155 represents a novel, robust and independent predictor of unfavorable prognosis for LPS patients. Multicentric external validation and thorough prospective analyses is required in order to robustly corroborate the prognostic significance of miR-155 in LPS. The previously reported oncogenic role of miR-155 in conjunction with our findings qualifies this miRNA as a potential therapeutic target for LPS. When the technology of microRNA silencing will be advanced [54] miR-155 could constitute the basis of a more optimized treatment for patients suffering from LPS.

## MATERIALS AND METHODS

### Liposarcoma and lipoma tissue samples: clinical and pathological features

A total of 83 FFPE tissue specimens from primary liposarcoma (LPS) (N=62) and lipoma (LPM) (N=21) patients who underwent curative resection, between 1990 and 2012 were included in our study. No neoadjuvant treatment had been administered in these patients. FFPE tissue samples along with detailed medical history, clinicopathologic characteristics and follow up survival information were obtained from the University of Ioannina Cancer Biobank Center (UICBC). The present research project was approved by the ethics committee of the University Hospital of Ioannina. All living patients gave written informed consent prior to study initiation. All diagnoses were reviewed by two experienced pathologists. Histological subtyping was based on the WHO classification of soft tissue tumors and tumor grade was calculated by the FNCLCC system [3].

The median age at diagnosis for LPS patients was 53.5 years (range: 20 – 86) and 61.3% were males, whereas for LPM patients was 47.5 years (range: 33 – 90) and 71.4% were males. The median tumor size was 10.0 cm for LPS and 4.65 cm for LPM patients. The distribution of LPS histologic subtypes were as follows: myxoid/round cell in 34 patients (54.8%), pleomorphic in 15 patients (24.2%), dedifferentiated in 9 patients (14.5%) well-differentiated in 4 patients (6.5%). The 38.1% of LPM tumors occurred in the trunk of the body and the rest in the extremities; for LPS the distribution of tumors was 62.9% in the extremities, 17.7% in the trunk and 19.4% were retroperitoneal.

Patients with positive surgical margins and/or harboring high-grade tumors were given adjuvant treatment, consisting of radiotherapy and/or chemotherapy. Median follow-up time of LPS patients was 73 months (2.0 – 215 months); median follow-up in patients still alive at the time of analysis was 109 months (33 – 187 months). During the follow-up period 33 patients died, 34 developed local recurrence and 10 progressed to metastatic disease.

The complete demographic, clinical and pathological characteristics of the patients are presented in details in Table 5.

**Table 5:** Clinicopathological and demographic characteristics of the LPS and LPM patients

Variable	
*LPS Patients (N = 62)*	
**Age (years)^a^**	53.5 (20.0 – 86.0)
**Tumor size (median, range cm)^a^**	10.0 (0.70 – 30.0)
**Follow-up time (months)^a^**	73.0 (2.0 – 215)
**Gender**	
Male	38 (61.3)
Female	24 (38.7)
**Location**	**N (%)**
Extremities	39 (2.7)
Trunk	11 (30.1)
Retroperitoneal	12 (53.4)
**Histologic Subtype**	**N (%)**
Well-differentiated	4 (6.5)
Dedifferentiated	9 (14.5)
Myxoid/Round Cell	34 (54.8)
Pleomorphic	15 (24.2)
**Grade FNCLCC**	**N (%)**
I	4 (6.5)
II	31 (50.0)
III	27 (43.5)
**TNM stage**	**N (%)**
IA	2 (3.2)
IB	3 (4.8)
IIA	9 (14.5)
IIB	22 (35.5)
III	25 (40.3)
IV	1 (1.6)
**Surgical Margins**	**N (%)**
Negative	36 (58.1)
Postitive	17 (27.4)
x	9 (14.5)
**Adjuvant Chemotherapy**	**N (%)**
No	47 (75.8)
Yes	15 (24.2)
**Adjuvant Radiotherapy**	**N (%)**
No	21 (33.9)
Yes	41 (66.1)
**Overall Survival**	**N (%)**
Alive	28 (45.2)
Deceased	33 (53.2)
x	1 (1.6)
**Relapse**	
No	27 (43.5)
Yes	34 (54.8)
x	1 (1.6)
**Metastatic Progression**	
No	51 (82.3)
Yes	10 (16.1)
x	1 (1.6)
***LPM Patients (N = 21)***	
**Age (years)^a^**	47.5 (33.0 – 90.0)
**Tumor size (cm)^a^**	4.65 (1.5 – 12.0)
**Gender**	**N (%)**
Male	15 (71.4)
Female	6 (28.6)
**Location**	**N (%)**
Extremities	8 (38.1)
Trunk	13 (61.9)

^a^ Median (minimum – maximum value).

x: Unknown.

### Homogenization of FFPE tissue specimens and total RNA extraction

A modified proteinase K incubation-Trizol treatment coupled protocol was used for the homogenization of FFPE tissue specimens. FFPE blocks were cut in 10-15 μm sections and 50 mg of them were transferred into Eppendorf tubes. Deparaffinization was performed with 1.0 mL xylene per sample, vortex and incubation in 50°C for 3 min in mild shaking in a Cooling ThermoMixer MKR 13 (HLC, Ditabis, Pforzheim, Germany). Samples were then centrifuged at full speed for 2 min in room temperature and the supernatant was discarded. The resulting pellet was washed two times from any residual xylene by 1 ml of 100% ethanol, vortex and centrifugation at full speed for 2 min. The sediment was incubated at 60°C for 3-10 min until complete ethanol evaporation. A total of 200 μL Lysis Buffer FL (Macherey-Nagel, Düren, Germany) and 20 μL proteinase k (20 mg/mL initial concentration) (New England BIolabs, Herts, UK) were added and samples were incubated overnight at 55°C in mild shaking. Subsequently, 100 μL of Decrosslink Buffer (Macherey Nagel, Düren, Germany) were added and incubation at 80°C for 15 min took place in order to terminate the proteinase k reaction and eliminate crosslinking of RNA. Samples were then cooled down in room temperature for 2 min, 1.0 mL of TRIzol® LS Reagent (Invitrogen, USA) was added and total RNA isolation was performed according to the manufacturer's instructions. RNA pellets were dissolved in RNA Storage Solution (Ambion, Austin, TX, USA) and stored in -80°C until further analysis. The concentration and purity of total RNA were determined in a BioSpec-nanospectrophotometer (Shimadzu, Kyoto, Japan).

### Polyadenylation of total RNA and reverse transcription

One μg of total RNA per sample was polyadenylated with the addition of 800 μM ATP and 1 U of *E. coli*Poly(A) Polymerase in the reaction buffer supplied by the manufacturer (New England Biolabs Inc., Ipswich, MA, USA) at 37°C for 60 min, followed by a reaction termination step at 65 °C for 10 min. Subsequently, the polyadenylated RNA was reverse transcribed with 100 U M-MLV reverse transcriptase (Invitrogen, USA) in the reaction buffer supplied by the manufacturer, in the presence of 20 U RNaseOUT™ recombinant ribonuclease inhibitor (Invitrogen, USA), and 0.25 μM poly(T) adapter(5’-GCGAGCACAGAATTAATACGACTCACTATAGGTTTTTTTTTTTTVN-3’) at 37°C for 60 min. An enzyme inactivation step followed at 70 °C for 15 min.

### Quantitative PCR for the expression analysis of candidate reference miRNAs and target miRNAs

A series of 14 qPCR assays were designed, developed and standardized for the expression analysis of 9 candidate reference miRNA molecules (miR-191, miR-103, miR-24, miR-28, miR-423, miR-16, miR-25, miR-331, miR-93) in order to find the most appropriate ones for expression normalization, as well as for 5 target molecules (miR-155, miR-21, miR-145, miR-143 and miR-451). The SYBR-Green-based qPCR assays were run in duplicates in 96-well fast reaction plates in 10 μL reactions (Applied Biosystems®, USA), consisting of Kapa SYBR® Fast Universal qPCR Master Mix (Kapa Biosystems) including Rox Low passive reference dye, a forward primer specific for each miRNA and a universal reverse primer all at a final concentration of 200 nM, as well as 1 ng of cDNA template. The sequences of all primers used are presented in Table 6. The 7500 Fast Real-Time PCR System (Applied Biosystems®, USA) following a rapid cycling thermal protocol consisting of a 3 min polymerase activation step at 95 °C and 40 cycles of denaturation – annealing/extension steps at 95 °C for 3 sec – 60 °C for 30 sec was used for all qPCR reactions, followed by a melting curve analysis step. A no-template control and a calibrator sample was included in each qPCR run which included duplicate reactions. The expression levels of the target miRNA molecules were calculated as Relative Quantification (RQ) units with the comparative C_T_ method (RQ = 2^-ΔΔCt^) *via* the 7500 software v.2.06 (Applied Biosystems®, USA) using two endogenous reference miRNAs (miR-191 and miR-103 combination) for normalization purposes.

**Table 6:** Sequences of the oligos used for qPCR

Oligo	Primer sequence (5’→3’)
miR-191 (F)	GAATCCCAAAAGCAGCTGAA
miR-103 (F)	CAGCATTGTACAGGGCTATGAAA
miR-25 (F)	ATTGCACTTGTCTCGGTCTGA
miR-16 (F)	TAGCAGCACGTAAATATTGGCG
miR-24 (F)	TGGCTCAGTTCAGCAGGAAC
miR-28 (F)	AAGGAGCTCACAGTCTATTGAGAA
miR-423 (F)	GGCAGAGAGCGAGACTTTAA
miR-93 (F)	CAAAGTGCTGTTCGTGCA
miR-331 (F)	GCCCCTGGGCCTATCCTA
miR-155 (F)	AATGCTAATCGTGATAGGGGTAA
miR-21 (F)	GTAGCTTATCAGACTGATGTTGAAA
miR-145 (F)	CCAGTTTTCCCAGGAATCCCTAA
miR-143 (F)	TGAGATGAAGCACTGTAGCTCAAA
miR-451 (F)	AAACCGTTACCATTACTGAGTTAA
Universal Reverse Primer	GCGAGCACAGAATTAATACGAC

F: Forward primer.

### Examining suitable molecules for normalization of miRNA expression in liposarcoma through a combined Genorm and NormFinder approach

A crucial step in gene expression analysis, including miRNA expression analysis, is the identification of suitable genes that could be used as reference for normalization purposes [37, 39]. It is generally acknowledged that there is no such thing as a general reference gene suitable for every type of tissue and under different conditions such as disease [38]. In LPS there has not been up to date a study to propose which genes could act as endogenous reference molecules for miRNA expression analysis. A widely accepted approach is to measure the expression of several candidate reference genes in a number of representative samples, and select the gene(s) that show least variation as reference(s) [37–39, 46]. For this reason, we chose to evaluate several endogenous reference miRNA molecules that are proposed in the literature in other cancer-related miRNA expression studies [38, 46, 55–62] in order to find the most suitable ones for LPS. We deliberately have not included larger RNA molecules or molecules of the SNORD/RNU family commonly used for miRNA normalization in fresh tissues, having in mind: i) the fact that it is preferable to use normalizers that chemically, structurally and biologically resemble as much as possible the target molecule [63], and ii) the extent of RNA degradation and chemical modifications that occurs in FFPE tissues [12, 13, 46] and could disturb more extensively molecules larger than miRNAs. Consequently, we evaluated the expression stability of 9 candidate reference miRNA molecules (miR-191, miR-103, miR-24, miR-28, miR-423, miR-16, miR-25, miR-331, miR-93) in a set of 22 LPS and LPM tissues, using the geNorm [39] and NormFinder [37] algorithms. Briefly, genNorm calculates and compares the M-value, a measure of variation of a gene compared to all other candidate genes, for all genes, eliminates the gene with highest M-value, and repeats the process until there is only two genes left. This last pair of genes remaining is proposed as the optimum combination of reference genes. NormFinder, in contrast to geNorm, takes into account information of groupings of samples, such as LPS *vs* LPM samples and calculates both intra- and inter- group variance and can also propose the single gene with most stable expression along with the best pair of genes with combined most stable expression [37–39].

### Quality control

The specificity, sensitivity and reproducibility of the developed qPCR assays were evaluated through quality control procedures consisting of: i) melting curve analysis coupled with 3.0% agarose gel electrophoresis for all amplicons that verified the presence of a unique peak in the melting curve and a unique band for each amplicon, respectively, ii) testing of several negative control samples such as no-template controls, reverse transcription-negative control and DNA template controls that led to an undetectable C_T_ signal in all cases, iii) the construction of standard curves for all miRNA molecules assayed, verifying that PCR efficiencies and linearity fell under the acceptable range for qPCR reactions, thus allowing calculation *via* the comparative C_T_ method and excluding the possibility of PCR inhibition, iv) estimating the assays’ reproducibility by analyzing a series of samples from different tumor parts in different qPCR runs and calculating the coefficient of variation from duplicate measurements [64].

Detailed information about the quality control procedures is described in Supplementary Table 1.

### Biostatistical analyses

Statistical significance between continuous variables of the study was tested by Spearman's correlation analysis. The differences in miRNA distribution across different nominal and ordinal variables of the study, such as the clinicopathological characteristics of LPS patients, were statistically tested by Mann-Whitney *U*, Kruskall-Wallis H or Jonckheere-Terpstra tests, where appropriate. The DeLong *et al* method was used for ROC curve analyses [65].

For the survival analyses, the expression levels of all target miRNA molecules included in the study were dichotomized according to the median expression value, thus avoiding the use of minimal P value statistics. Consequently, LPS patients were stratified into miR-high and miR-low individuals for each miRNA. The subsequent survival analyses included the generation of Kaplan-Meier overall-, relapse free-, and metastatic progression free- survival (OS, RFS, PFS) curves, and the development of Cox proportional hazard regression models for the evaluation of the prognostic potential of miR-155, miR-21, miR-145 and miR-451 expression for LPS patients. A full multivariate model was developed including important demographic/clinical factors and currently used strong prognostic indicators for LPS, such as tumor grade, tumor size, tumor location, surgical margins, age, gender etc. Alternative multivariate models were constructed in order to avoid including concurrently in the same model important yet highly correlated indicators, such as surgical margins and tumor location and thus restricting collinearity phenomena that could negatively affect the prognostic models’ accuracy.

All statistical analyses were performed using the IBM Statistics v.23.0 and the MedCalc v.12.5 software. Two-tailed tests were used and P values < 0.05 were adapted for statistical significance.

This work was supported by a research grant of the Hellenic Society of Medical Oncology.

## SUPPLEMENTARY MATERIALS FIGURES AND TABLES


