# Supplementary material for: Assessing the clinical value of microRNAs in formalin-fixed paraffin-embedded liposarcoma tissues: Overexpressed miR-155 is an indicator of poor prognosis
Source: Oncotarget. 2016 Dec 28;8(4):6896–913. doi: 10.18632/oncotarget.14320 (PMC5351678; doi:10.18632/oncotarget.14320)
Supplement: Supplementary file 1 [file oncotarget-08-6896-s001.pdf]

Assessing the clinical value of microRNAs in formalin-fixed paraffin-embedded liposarcoma tissues: Overexpressed miR-155 is an indicator of poor prognosis

SUPPLEMENTARY FIGURE AND TABLE

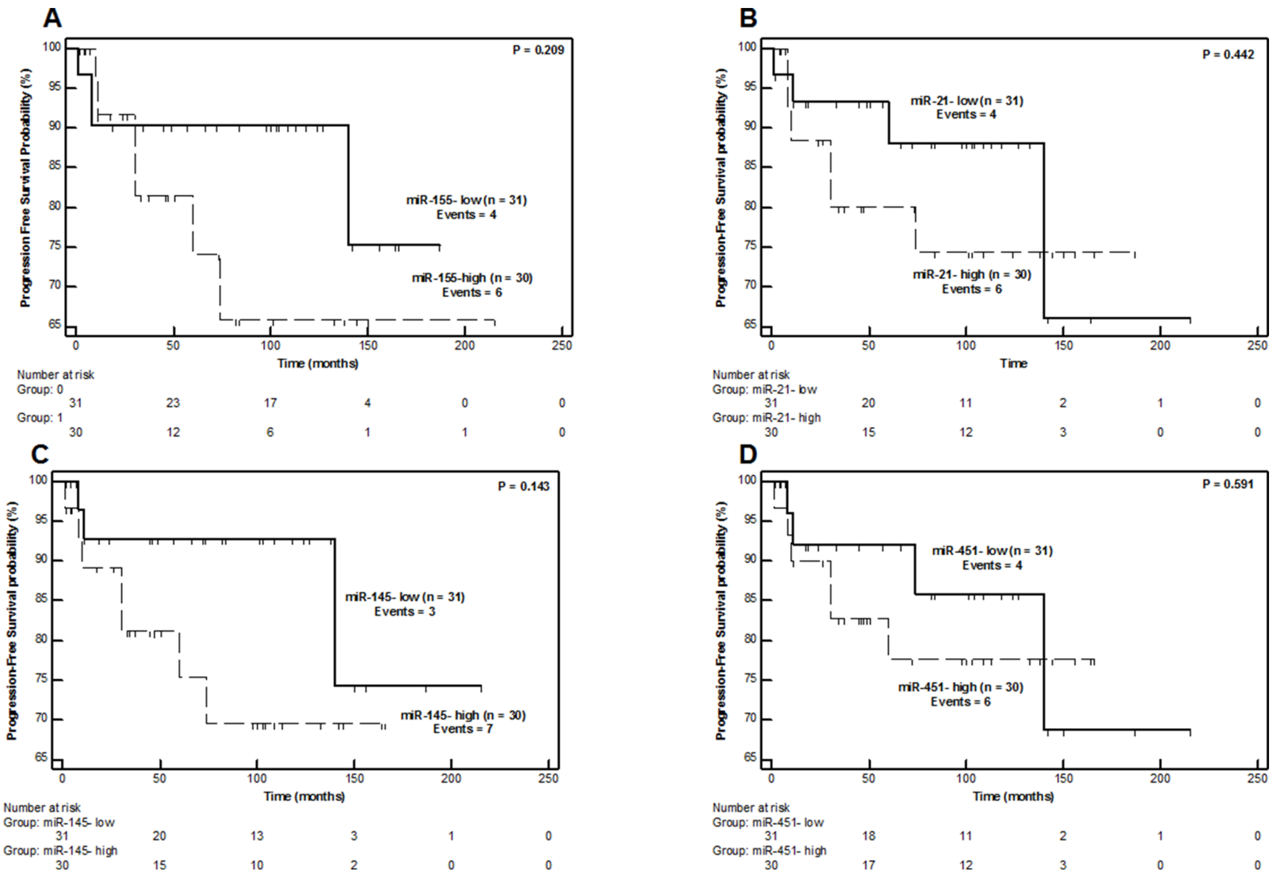

Supplementary Figure 1: Progression-free Survival Kaplan-Meier Curves for miR-155 **(Part A)**, miR-21 **(Part B),** miR-145 **(Part C)** and miR-451 **(Part D)** expression in liposarcoma patients. P values calculated by the log-rank algorithm.

Supplementary Table 1: Quality control data of the qPCR assays developed

qCPR Assay	r^2 of standard curve	Reaction efficiency	ng of cDNA included in standard curve	Product Tm (Melting Curve)	% Coefficient of Variation (CV)*
miR-155	0.9984	98.73%	0.001-10	73.78	9.94
miR-21	0.9988	95.36%	0.0001-10	73.18	14.3
miR-145	0.9995	90.45%	0.0001-10	73.48	7.76
miR-143	0.9993	90.68%	0.0001-10	74.23	16.2
miR-451	0.9987	93.06%	0.0001-10	72.58	15.6
miR-191	0.9997	91.69%	0.0001-10	74.08	N/A
miR-103	0.9999	90.66%	0.001-10	73.93	N/A
miR-25	0.9995	90.97%	0.001-10	74.74	N/A
miR-16	0.9993	91.18%	0.001-10	74.23	N/A
miR-24	0.9997	98.96%	0.001-10	74.89	N/A
miR-28	0.9984	93.65%	0.001-10	74.34	N/A
miR-423	0.9997	92.39%	0.001-10	74.19	N/A
miR-93	0.9984	96.98%	0.001-10	75.04	N/A
miR-331	0.9997	95.04%	0.001-10	74.74	N/A

N/A: Not applicable.

*Coefficient of variation (CV) from duplicate measurements refers to the normalized expression levels of miR-155, miR-21, miR-145, miR-143 and miR-451.
